# miR-1-3p downregulation drives tumor invasion in oral squamous cell carcinoma: insights from *in vitro*, *in vivo*, and *ex vivo* models

**DOI:** 10.1007/s11033-026-11914-8

**Published:** 2026-05-14

**Authors:** Jessica Boscariol da Silva, José Leonardo de Oliveira, Gilberto Mendes Menderico, Laura Sichero, Hernandes F. Carvalho, Luiz Paulo Kowalski, Leandro Luongo Matos

**Affiliations:** 1 Faculdade de Medicina, Programa de Pós-Graduação em Anestesiologia, Ciências Cirúrgicas e Medicina Perioperatória, Department of Surgery, Universidade de São Paulo, São Paulo, SP Brazil; 2 Postgraduate Program in Molecular and Morphofunctional Biology – (UNICAMP), Department of Structural and Functional Biology, University of Campinas (UNICAMP), Campinas, SP Brazil; 3 Head and Neck Surgery Department - Laboratório de Investigação Médica 28 (LIM28), Department of Surgery, University of São Paulo Medical School, Av. Dr. Eneas de Carvalho Aguiar, 255, 8th floor, room 8174, SP 05403-000 Sao Paulo, Brazil; 4 Faculdade de Medicina, Instituto do Cancer do Estado de Sao Paulo ICESP, Hospital das Clinicas HC FMUSP, Universidade de Sao Paulo, Sao Paulo, São Paulo, Brazil; 5 Comprehensive Center for Precision Oncology, Department of Oncology and Radiotherapy, Universidade de Sao Paulo, São Paulo, SP Brazil; 6 Department of Structural and Functional Biology, University of Campinas (UNICAMP), Campinas, SP Brazil; 7 Head and Neck Surgery, Department of Surgery, Faculdade de Medicina Universidade de São Paulo, São Paulo, SP Brazil; 8 Faculdade Israelita de Ciências da Saúde Albert Einstein, São Paulo, SP Brazil

**Keywords:** Mouth neoplasms, MicroRNAs, Neoplasm invasion, Zebrafish xenograft, Biomarkers, Tumor

## Abstract

**Aim:**

Oral squamous cell carcinoma (OSCC) often presents as an invasive tumor with poor prognosis. Recent evidence suggests that microRNAs, particularly miR-1-3p, may regulate the molecular pathways associated with tumor invasion. This study aimed to investigate the functional role of miR-1-3p in OSCC invasion using integrated *in vitro*, *in vivo*, and *ex vivo* approaches, and to evaluate its expression in tumors with varying depths of invasion (DOI).

**Methods:**

SCC-9 cells were transfected with miR-1-3p mimic and inhibitor. Invasion was assessed using transwell assays. Zebrafish xenografts were generated to evaluate tumor area, invasion depth, migration, and metastasis. Additionally, miR-1-3p expression was quantified by RT-qPCR in 26 human OSCC samples stratified by different groups of DOI.

**Results:**

miR-1-3p inhibition significantly increased tumor cell invasion *in vitro* (*P* = 0.039) and resulted in larger, deeper, and more migratory tumors *in vivo* (*P* < 0.05). All *in vitro* experiments were performed in duplicate, which may limit the precision of quantitative estimates. Metastases were observed in 30.8% of larvae injected with miR-1-3p–inhibited cells, representing a trend toward increased metastatic behavior (*P* = 0.052). In human samples, miR-1-3p expression was significantly negatively correlated with DOI (Spearman’s *R* = − 0.435; *P* = 0.030), and deeply invasive tumors exhibited lower expression levels (*P* = 0.040).

**Conclusion:**

This integrative analysis supports an association between miR-1-3p downregulation and increased invasive tumor behavior in OSCC across experimental models and human tumor samples. These findings provide preliminary translational evidence and support further investigation into the potential role of miR-1-3p as a biomarker in OSCC.

## Introduction

Head and neck cancers encompass a heterogeneous group of tumors arising from various anatomical sites, of which approximately 90% are squamous cell carcinomas (HNSCC) [1]. These malignancies represent a significant global public health concern because of their substantial morbidity and mortality. HNSCC is the sixth most common cancer worldwide [2]. The primary risk factors associated with HNSCC include tobacco use, alcohol consumption, and human papillomavirus (HPV) infection [3].

Oral squamous cell carcinoma (OSCC) accounts for approximately 40% of all HNSCC cases [4]. Compared to other HNSCCs, patients with OSCC generally have a poorer prognosis, largely due to the aggressive invasive behavior of the tumor and frequent diagnosis at advanced stages [5]. Several factors have been associated with unfavorable prognosis. Among these, depth of invasion (DOI) has recently gained attention as a robust prognostic marker and has been incorporated into the current TNM staging system [6]. Clinically, DOI serves as a surrogate for a tumor’s invasive capacity, suggesting an underlying biological process potentially governed by molecular mechanisms.

The initiation and progression of tumor invasion in OSCC is a multistep process that involves cumulative genetic, epigenetic, and transcriptomic alterations. MicroRNAs (miRNAs) have emerged as key regulators of tumor biology [7]. Increasing evidence supports the role of miRNAs in modulating extracellular matrix (ECM) degradation and remodeling, which are crucial steps in the invasive cascade [8, 9]. Although the precise molecular mechanisms remain unclear, several miRNAs have been implicated in this regulatory network. In OSCC, both upregulated and downregulated miRNAs are associated with disease progression [10]. In a previous study conducted by our group using formalin-fixed paraffin-embedded (FFPE) OSCC samples, we found that reduced expression of miR-1-3p and miR-133a-3p, along with elevated levels of miR-21-5p, were associated with deeper tumor invasion and ECM remodeling. In the same study, we also found that lower expression of miR-1-3p was associated with higher levels of metalloproteinase 2 (MMP-2) in fibroblasts associated with the tumor, which in turn was linked to basement membrane degradation and promotion of the tumor invasion process [8].

Given the demonstrated role of miR-1-3p in the tumor invasion process through basement membrane degradation mediated by increased MMP-2 expression in tumor-associated fibroblasts, the objective of this study was to investigate the role of miR-1-3p in the biological mechanisms underlying tumor invasion using experimental models. Additionally, we sought to validate the differential expression of these miRNAs in a cohort of patients with invasive OSCC to support a translational research approach.

## Methods

### Experimental models

The experimental study consisted of *in vitro* invasion assays and *in vivo* tumor formation in a zebrafish model. All animal experiments were approved by the Animal Ethics Committee (*Comitê de Ética no Uso de Animais* – CEUA) of the Faculdade de Medicina da Universidade de São Paulo – FMUSP (protocol no. 1538/2020) and were conducted in accordance with the institutional guidelines and national regulations for the care and use of laboratory animals.

#### Cell line and culture

We used a commercially available cell line from the American Type Culture Collection (ATCC) derived from squamous cell carcinoma of the tongue (SCC-9 – CRL-1629). Cells were maintained in 25 cm² flasks (Corning^®^, New York, USA) at 37 °C in a 5% CO₂ incubator, using DMEM: Ham F12 culture medium supplemented with 1.2 g/L sodium bicarbonate, 2.5 mM L-glutamine, 15 mM HEPES, and 0.5 mM sodium pyruvate, and further supplemented with 400 ng/mL hydrocortisone (BCRJ^®^, Rio de Janeiro, Brazil). All procedures related to cell culture were performed under a vertical laminar flow hood that had been previously disinfected with 70% ethanol and exposed to UV light for 20 min. Upon reaching 80% confluence, cells were subcultured using trypsin and maintained in culture for subsequent experiments.

#### Selected microRNAs, mimics and inhibitors

miR-1-3p, previously identified by our group as differentially expressed in deeply invasive versus superficial oral tumors through miRNA array analysis [8], was evaluated. Both mimics and inhibitors of miR-1-3p were synthesized by Thermo Fisher Scientific^®^ (Waltham, MA, USA) and used at the time of transfection [11]. Briefly, mirVana miRNA mimics are chemically modified, double-stranded RNA molecules designed to mimic endogenous miRNAs, resulting in the negative regulation of target mRNA translation through sequestration or degradation. The mature (guide) strand is incorporated by Argonaute proteins (Ago), directing gene silencing, whereas the passenger strand may or may not have functional targets. Chemical modifications in the passenger strand improve the uptake of the guide strand by Ago. These mimics were optimized for maximal effects at low concentrations (0.3 nM). In contrast, mirVana miRNA inhibitors are chemically modified single-stranded oligonucleotides designed to bind and inhibit endogenous miRNAs, resulting in positive regulation of their mRNA targets.

#### Transfection and RNA processing

SCC-9 cells at 80% confluence were transfected with Lipofectamine^®^ RNAiMAX (Thermo Fisher Scientific^®^, Waltham, MA, USA) according to the manufacturer’s instructions (MH10617, MH10206, MH10413, MC10617, MC10206, MC10413, 4464058, 4467046, 4464062, 4464080, and 13778030). For each well in a 24-well plate, 3 µL of transfection reagent was diluted in 50 µL of OPTI-MEM I Reduced Serum Medium (Invitrogen, Carlsbad, CA, USA), 10 µM of oligonucleotide was diluted in 50 µL of the same medium, and 5 × 10⁴ to 1 × 10⁵ cells per well, depending on the experiment. The complexes were incubated for 5 min at room temperature before being added to 500 µL of F12 medium per well (final volume, 600 µL). RNA was extracted at 48 h post-transfection. This protocol has been used for both miRNA mimics and inhibitors [12]. Positive and negative controls were also used. For mimics, miR-1 served as a positive control because it is known to downregulate Protein Tyrosine Kinase 9 (PTK9), which was monitored to confirm its activity [13]. For inhibitors, let-7c was used to monitor the increase in High Mobility Group AT-hook 2 (HMGA2) mRNA expression [14]. Non-targeting sequences with no detectable effects were used as negative controls. RT-PCR was used to assess the efficacy of all the constructs.

#### RNA extraction

RNA was extracted using TRIzol^®^ (Invitrogen, USA). After transfection, samples were processed with TRIzol^®^, chloroform extraction, and isopropanol precipitation, followed by centrifugation. RNA pellets were washed three times with 75% DEPC ethanol, dried, resuspended in 15 µL of Milli-Q water, and stored at − 80 °C. RNA concentration was measured by NanoDrop Lite (Thermo Fisher Scientific^®^, USA) and standardized to 10 ng/µL for downstream analyses.

#### cDNA synthesis

cDNA synthesis was performed using two kits: the High-Capacity cDNA Reverse Transcription Kit (Thermo Fisher Scientific^®^, Waltham, MA, USA) for positive controls (miR-1 and let-7c) and the TaqMan^®^ MicroRNA Reverse Transcription Kit for experimental miRNAs. Reactions using the first kit were carried out in a final volume of 20 µL with the following thermal profile: 10 min at 20 °C, 120 min at 37 °C, 85 min at 5 °C, and hold at 4 °C. For the second kit, a final volume of 15 µL was used, with 30 min at 16 °C, 30 min at 42 °C, and 5 min at 85 °C, and held at 4 °C.

#### RT-PCR

**Fig. 1:**
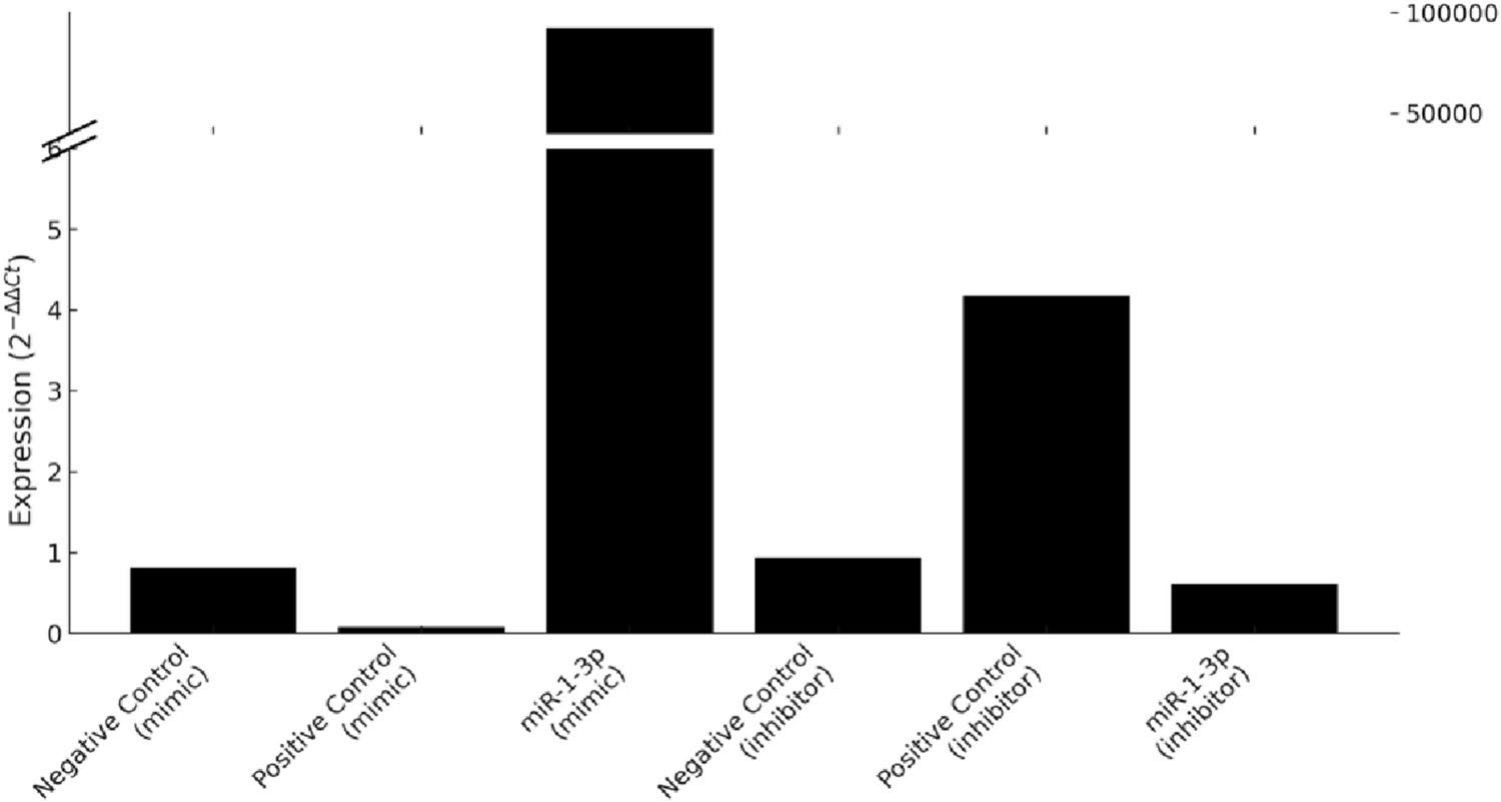
Validation of miR-1-3p modulation following transfection (**A**). Expression levels of miR-1-3p after transfection were assessed using the 2^–ΔΔCt^ method. In the mimic group, transfection with miR-1-3p mimic resulted in marked overexpression of miR-1-3p compared to both positive and negative controls. Conversely, transfection with miR-1-3p inhibitor led to decreased expression compared to the positive control for inhibition. *Note*: Bars represent individual experimental groups; broken y-axis was applied to accommodate the highly increased expression observed in the mimic-transfected cells. *In vitro* effect of miR-1-3p modulation on tumor invasion

For qPCR, 5 µL of cDNA was used with 10 µL of TaqMan^®^ Fast Advanced Master Mix 2x, 4 µL RNase-free water, and 1 µL of specific probes (TaqMan^®^ Advanced miRNA Assay 20x or TaqMan™ Gene Expression Assay FAM 20x). The following miR-1-3p primer sequence was used: UGGAAUGUAAAGAAGUAUGUAU. Reactions (20 µL) were run on an Applied Biosystems 7300/7500 Real-Time PCR System under the following conditions: 95°C for 10 min, followed by 40 cycles of 95°C for 15 s and 60°C for 1 min. Controls included U6snRNA (forward: 5′-CTTTGGTATCGTGGAAGGACTC-3′, reverse: 5′-GTAGAGGCAGGGATGATGTTCT-3′) and GAPDH (forward: 5′-GCTTCGGCAGCACATATACTAAAAT-3′, reverse: 5′-CGCTTCACGAATTTGCGTGTCAT-3′)[15]. All experiments were performed in duplicate. The results were analyzed using the 2^–ΔΔCt^ method, and the inhibition and mimetization of SCC-9 cells are shown in Fig. 1.

#### Invasion assay

Cell invasion capacity was assessed using 8 μm pore-size Transwell inserts (Corning^®^, NY, USA) coated with Matrigel (Corning^®^ Matrigel^®^, NY, USA). miRNA transfected SCC-9 cells (2.1 × 10⁴) were seeded in the upper chamber in serum-free medium, while the lower chamber contained F12 medium with 10% fetal bovine serum as a chemoattractant. The plates were then incubated at 37 °C and 5% CO₂ for 48 h. Non-invading cells were removed, and invading cells were fixed with 4% paraformaldehyde and stained with 0.1% crystal violet. The inserts were analyzed under 20× magnification in six fields per insert. All experiments were performed in duplicate [16, 17].

#### *In vivo* assay

##### Zebrafish xenograft

SCC-9 cells were transduced to express tdTomato fluorescent protein and blasticidin resistance gene (Tomato-BSD) [18]. These cells were xenografted into Danio rerio AB and Tg(Fli: GFP) embryos 48 h post fertilization (hpf), and a pool of 50 embryos per group was created [19, 20]. The experimental groups included embryos injected with miRNA-transfected SCC-9 cells, and a control group injected with non-transfected tumor cells.

Zebrafish embryos were incubated in E3 embryo medium (NaCl 5 mM, KCl 0.17 mM, CaCl₂ 0.33 mM, MgSO₄ 0.33 mM, pH 7.0) and kept at 28 °C. Embryonic and larval stages are expressed as hours post-fertilization (hpf) or days post-fertilization (dpf) [21]. Adult zebrafish were housed at the Universidade Estadual de Campinas (UNICAMP, Campinas, São Paulo, Brazil) using the Aquatic Habitats Z-Hab (Irland) system. Animals were kept at 28 °C ±, pH 7.3 ± 0.2, conductivity 500–800 µS, under a 14 h/10 h light/dark cycle, and fed thrice daily (Tetra ColorBit, Blacksburg, EUA) [22]. One week before mating, the males and females were separated to synchronize their spawning. Three days before microinjection, mating pairs (two males and one female) were transferred to breeding tanks and embryos were collected the following morning. Microinjections were performed at 48 hpf using borosilicate capillaries pulled with a PUL-1000 (WPI). Labeled cells were suspended in PBS at 1 × 10^5^ cells/µL and injected into the perivitelline space (PVS) under a stereomicroscope (Axio Zoom.V16 Carl Zeiss). Embryos were placed on a 1,5% agarose plate to ensure stability [22, 23]. Twenty-four hours post-injection, the larvae were anesthetized with 0.168 mg/L tricaine, embedded in agarose, and imaged using an EVOS fluorescence microscope (Thermo Fisher, USA). At the end of the experiment, the larvae were euthanized with 4.2 mg/L tricaine and fixed in 4% paraformaldehyde. Tumor burden was assessed by imaging and measuring the (1) tumor area, (2) depth of invasion relative to larval width, and (3) presence of distant migration/metastasis in each group.

### *Ex vivo* validation study

The *ex vivo* study was approved by the Institutional Review Board of the Hospital das Clínicas, Faculdade de Medicina, Universidade de São Paulo (CAPPesq - *Comissão de Ética para Análise de Projetos de Pesquisa HCFMUSP*) under protocol number 228/14 (CAAE 32884214.5.0000.0065). All procedures involving human participants were conducted in accordance with the ethical standards of the institutional research committee and with the Declaration of Helsinki. Written informed consent was obtained from all participants prior to inclusion in the study. The study comprised a retrospective cohort of individuals diagnosed with oral squamous cell carcinoma (OSCC) who underwent surgical treatment by the Head and Neck Surgery team at the Instituto do Câncer do Estado de São Paulo, Hospital das Clínicas, Faculdade de Medicina, Universidade de São Paulo (ICESP-HCFMUSP) between 2009 and 2015. To efficiently assess the relationship between depth of invasion (DOI) and molecular markers and considering the impracticality of analyzing the entire cohort of approximately 600 patients, tissue specimens from consecutive patients presenting with fixed DOIs of 5 mm, 15 mm, and 25 mm were selected. These specific DOI thresholds were chosen to represent superficial, moderate, and deep invasion while minimizing selection bias.

A total of 26 patients were included in the study. This retrospective design allowed the inclusion of all available cases corresponding to predetermined DOI values. Clinical, demographic, and pathological data were retrieved from the institution’s electronic health records. DOI was reassessed on hematoxylin and eosin-stained slides by a pathologist blinded to the clinical data. The DOI was measured from the basement membrane to the deepest point of tumor infiltration [24]. The same pathologist was responsible for selecting FFPE blocks for subsequent molecular analyses.

#### MicroRNA expression analysis

miRNA expression levels were quantified by reverse transcription and quantitative polymerase chain reaction (RT-qPCR). Total RNA was isolated from ten 5-µm sections of formalin-fixed, paraffin-embedded (FFPE) tumor samples using the MagMAX FFPE RNA Ultra Kit (Applied Biosystems^®^, Foster City, CA, USA), following the manufacturer’s instructions. RT-qPCR assays were conducted using the TaqMan Low Density Array platform (Thermo Fisher Scientific, Waltham, MA, USA). RNA samples were reverse-transcribed with polyadenylation to synthesize complementary DNA (cDNA), as specified by the manufacturer. The miRNA primer sequences used were UGGAAUGUAAAGAAGUAUGUAU for miR-1-3p and for miR-let-7i UGAGGUAGUAGUUUGUGCUGUU. Each sample was analyzed in triplicate, and cycle threshold (Ct) values were obtained using the Thermo Fisher Cloud platform. miRNAs with Ct values exceeding 38 were deemed unreliable and excluded from further analysis. Expression data were normalized across samples using the quantile normalization method implemented in Expander software, and miR-let-7i was used as the endogenous reference, as determined in a previous study by our group [8]. Relative quantification of miRNA expression was performed using the ΔΔCt method to identify the up-regulation or down-regulation of specific targets.

### Statistical analysis

Descriptive statistics for continuous variables were presented as means accompanied by either standard deviation (SD) or standard error (SE). Mean ranks were used to present the results of some quantitative variables. Categorical variables are summarized using absolute counts and corresponding percentages. The distribution of continuous variables was assessed using the Kolmogorov-Smirnov test, and all variables were classified as non-parametric. The Mann-Whitney U test was used to compare two independent groups. For comparisons involving three or more groups, Kruskal-Wallis’ test followed by Dunn’s post hoc test was performed. Associations between categorical variables and group distributions were examined using the chi-squared test or Fisher’s exact test, as appropriate. Spearman’s rank correlation and linear regression analyses were used to assess the association between two continuous variables. Receiver operating characteristic (ROC) curve analysis was performed to evaluate the discriminative ability of continuous variables, and the area under the curve (AUC) was calculated. Cutoff values were studied based on Youden’s index. Survival outcomes were analyzed using the Kaplan–Meier method, and differences between groups were assessed using the log-rank test. Given the multi-level design of the study, analyses were performed across distinct experimental contexts (*in vitro*, *in vivo*, and *ex vivo*), each addressing related but non-identical biological questions. Therefore, tests were not treated as belonging to a single inferential family, and no formal multiple comparison correction was applied. Instead, results were interpreted within an exploratory, hypothesis-generating framework, emphasizing consistency of direction and effect across independent models rather than isolated statistical significance. All statistical analyses were performed using IBM SPSS Statistics (version 26.0; IBM^®^, Endicott, New York, USA).

## Results

### *In vitro* analyses

**Fig. 2:**
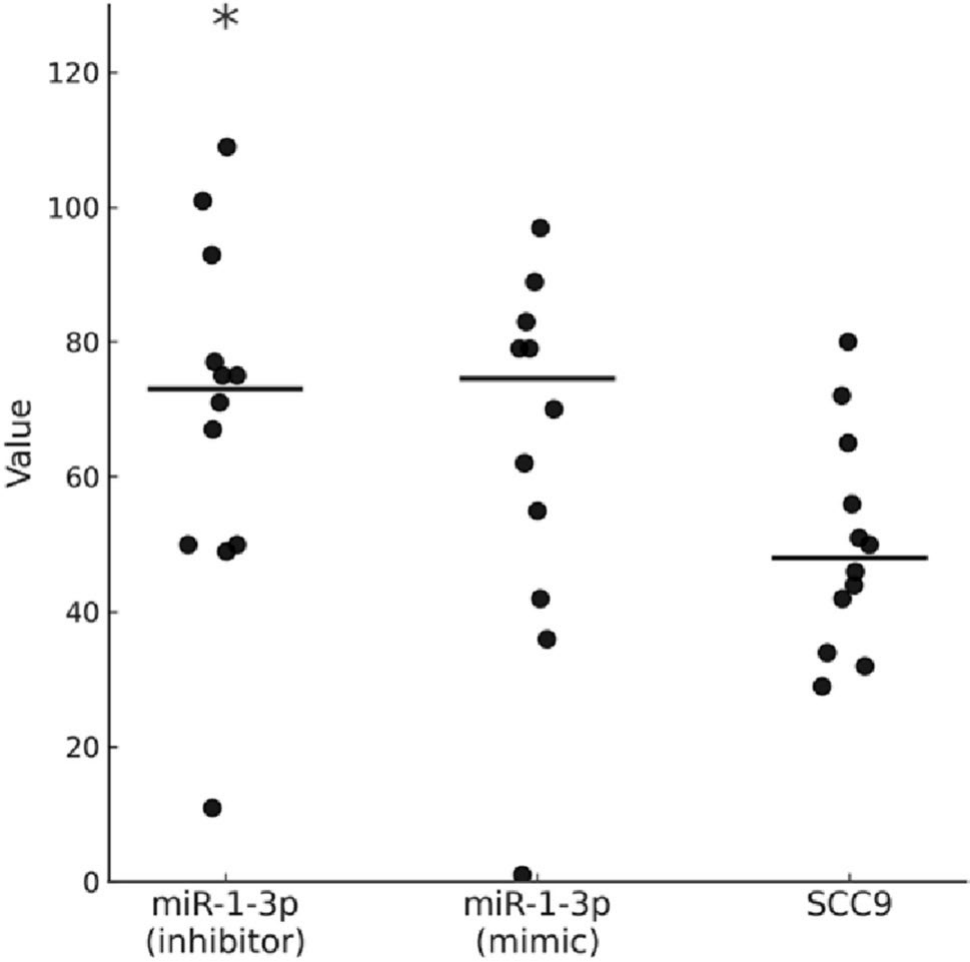
Impact of miR-1-3p inhibitor and mimic upon the invasion potential of SCC-9 cells. Inhibition of miR-1-3p resulted in significantly increased invasion compared to the control group (miR-1-3p inhibitor: mean rank = 14.8; mean = 69; SE = 7.7 vs. SCC-9: mean rank = 9.5; mean = 50.1; SE = 4.8; *P* = 0.039; Mann–Whitney U test). Mimic-induced overexpression of miR-1-3p did not significantly alter invasion compared to controls (miR-1-3p mimic: mean rank = 15.6; mean = 70; SE = 10.4 vs. SCC-9: mean rank = 9.9; mean = 60; SE = 5.9; *P* = 0.068; Mann–Whitney U test). *Note*: Horizontal lines represent group medians. Asterisks indicate statistically significant difference (*P* < 0.05)

To investigate the functional role of miR-1-3p in tumor invasion, *in vitro* assays were performed following both inhibition and overexpression of this miRNA. Inhibition of miR-1-3p significantly increased the invasive potential of SCC-9 tumor cells compared with that of the control group (*P* = 0.039). In contrast, mimic-induced overexpression of miR-1-3p did not result in a statistically significant reduction in cell invasion (*P* = 0.068), as shown in Fig. 2. Importantly, all *in vitro* assays were performed in duplicate, and therefore quantitative estimates should be interpreted with caution due to limited experimental replication.

### *In vivo* analyses

**Fig. 3:**
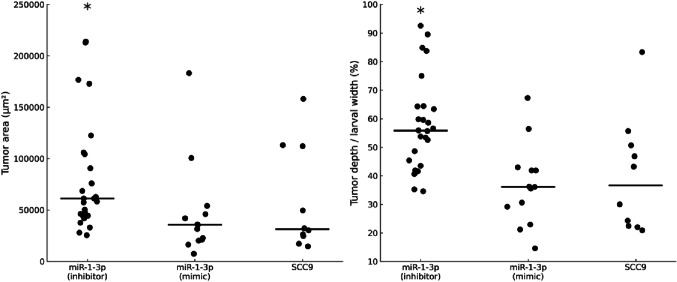
*In vivo* effects of miR-1-3p modulation in the zebrafish xenograft model. Tumor area (in square micrometers) was significantly increased in the miR-1-3p inhibition group (mean rank = 21; mean = 93,130.3; SE = 11,271.3) compared to the control group SCC-9 (mean rank = 12.1; mean = 57,883.2; SE = 16,042; *P* = 0.023; Mann–Whitney U test). No significant difference was observed in the mimic group (mean rank = 11.6; mean = 47,512.1; SE = 13,026.6; *P* = 0.784). The proportion of tumor depth relative to larval width was also significantly higher in the inhibition group (mean rank = 21.1; mean = 56.7%; SE = 3.2) compared to SCC-9 (mean rank = 11.7; mean = 40%; SE = 6.3; *P* = 0.015), whereas the mimic group showed no significant difference (mean rank = 11.7; mean = 36.7%; SE = 4; *P* = 0.832). *Note*: Horizontal bars represent medians. Asterisks indicate statistically significant differences (*P* < 0.05)

To further explore the functional role of miR-1-3p in tumor behavior, *in vivo* assays were performed using a zebrafish xenograft model. Inhibition of miR-1-3p resulted in a significant increase in tumor area compared to the SCC-9 control group (*P* = 0.023), whereas mimic-induced overexpression had no significant effect on tumor size (*P* = 0.784). The proportion of tumor depth relative to larval width was also significantly greater in the inhibition group (*P* = 0.015), with no significant difference observed following mimic treatment (*P* = 0.832) (Fig. 3).

**Fig. 4:**
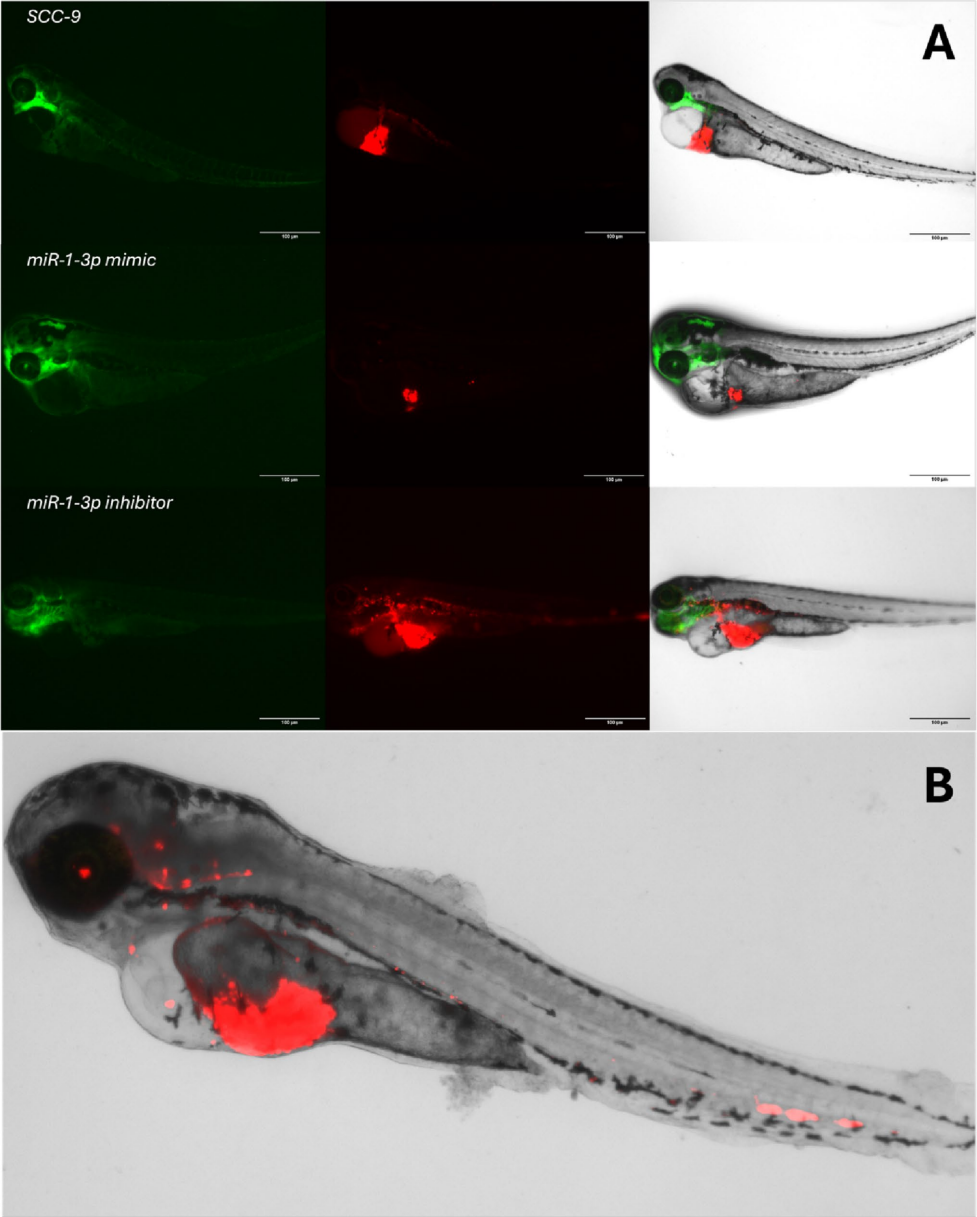
Representative fluorescence microscopy images of Tg(Fli: GFP) zebrafish larvae xenografted with SCC-9 cells transfected with either control, miR-1-3p mimic, or miR-1-3p inhibitor. (**A**) Left column: green fluorescence showing the anatomy of the larvae. Middle column: red fluorescence highlighting the human tumor cells. Right column: merged images demonstrating tumor localization and spread. Larvae injected with miR-1-3p inhibitor exhibited a more extensive tumor mass in the abdominal region and disseminated tumor foci toward the head and blood vessels of the tail, consistent with increased local invasion and metastatic behavior. In contrast, larvae injected with miR-1-3p mimic cells showed smaller and more localized tumors. The SCC-9 control group presented intermediate tumor size and distribution. Scale bars: 100 μm. (**B**) Representative image of an AB zebrafish larva (1dpi) with SCC-9 tumor cells transfected with a miR-1-3p inhibitor. The larva shows a large, deeply invasive primary tumor in the ventral region, as well as disseminated metastatic foci in both the head and tail

Additional analyses were performed to assess the impact of miR-1-3p modulation on tumor behavior in a zebrafish model. In the inhibition setting, metastases were observed in 30.8% of the larvae injected with miR-1-3p inhibitor-transfected cells (8/26), whereas no metastases were observed in the SCC-9 control group (0/10). This difference approached statistical significance (*P* = 0.052, Fisher’s exact test), suggesting a trend toward increased metastatic behavior associated with miR-1-3p inhibition. Migration was more frequent in the miR-1-3p inhibition group, occurring in 96.2% of cases (25/26) compared to 50% (5/10) in the SCC-9 controls (*P* = 0.003). In the mimic condition, metastases were identified in 15.4% (2/13) of the larvae injected with miR-1-3p mimic-transfected cells, while no metastases were observed in the SCC-9 group (0/10), with no statistically significant difference (*P* = 0.486). Nonetheless, migration was present in all larvae injected with miR-1-3p mimics (100%, 13/13) compared to 50% in the SCC-9 group (5/10), a statistically significant difference (*P* = 0.007, Fisher’s exact test). A representative image of a zebrafish larva injected with miR-1-3p–inhibited tumor cells showed a large, deeply invasive primary tumor in the ventral region, along with evident metastatic foci in the head and tail regions (Fig. 4).

### *Ex vivo* validation study

**Fig. 5:**
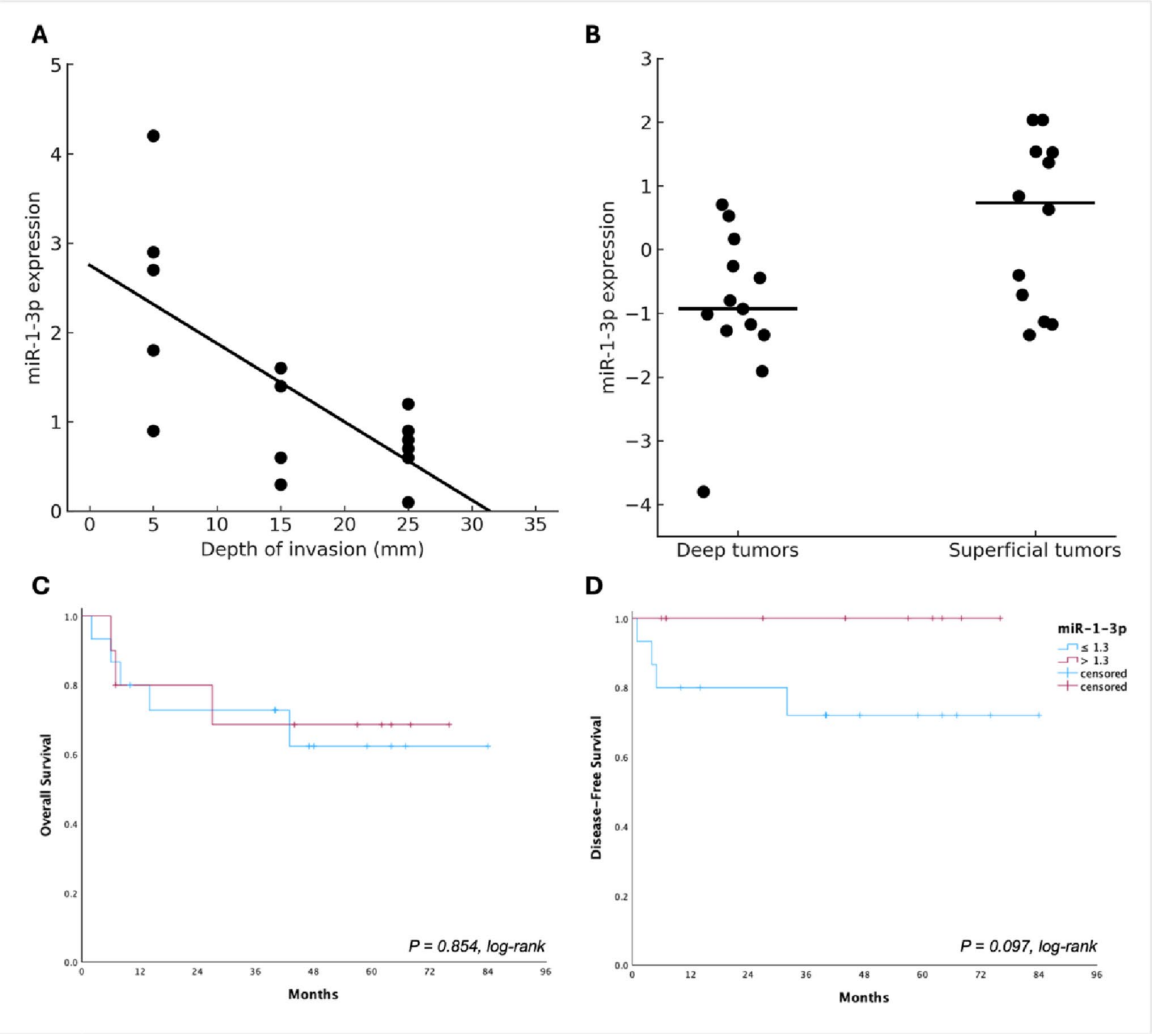
Association between miR-1-3p expression and tumor invasion and survival outcomes in oral squamous cell carcinoma. (**A**) A significant, but moderate, negative correlation was observed between miR-1-3p expression and depth of invasion (DOI) (Spearman’s *R* = − 0.435; 95% CI: − 0.714 to − 0.035; *P* = 0.030), indicating that deeper tumors tend to express lower levels of miR-1-3p. The linear regression curve illustrates this inverse relationship (R² = 0.261; *P* = 0.009). (**B**) Direct comparison between deeply and superficially invasive tumors confirmed significantly lower miR-1-3p expression in the former group (deep tumors: mean rank = 10.1; mean = 0.7; SE = 0.1 vs. superficial tumors: mean rank = 16.1; mean = 1.89; SE = 0.4; *P* = 0.040, Mann–Whitney U test). (**C**) Kaplan–Meier analysis of overall survival according to miR-1-3p expression levels (cutoff: 1.3) showed no significant difference between groups (log-rank *P* = 0.854), with estimated 5-year OS rates of approximately 62% in the low-expression group and 68% in the high-expression group. (**D**) Kaplan–Meier analysis of disease-free survival demonstrated a non-significant trend toward worse outcomes in patients with lower miR-1-3p expression (log-rank *P* = 0.097), with estimated 5-year DFS rates of approximately 72% in the low-expression group and 100% in the high-expression group. *Note*: Each dot represents one tumor sample. The black line in Panel A represents the linear regression fit. The horizontal bars in panel B indicate the medians

To explore the relationship between tumor depth and miR-1-3p expression, patients were stratified into two groups: 12 tumors with superficial invasion (DOI = 5 mm) and 14 tumors with deeper invasion (6 cases with DOI = 15 mm and 8 cases with DOI = 25 mm). A significant, but moderate, negative correlation was observed between miR-1-3p expression and the depth of invasion (Spearman’s *R* = − 0.435; 95% CI: − 0.714 to − 0.035; *P* = 0.030). This inverse relationship was further supported by the linear regression analysis (R² = 0.261; *P* = 0.009; Fig. 5A). To complement the correlation and regression findings, a direct comparison of miR-1-3p expression between deep and superficial tumors was performed. Tumors with greater DOI exhibited significantly lower miR-1-3p expression levels than those with superficial tumors (*P* = 0.040; Fig. 5B). ROC curve analysis was performed to evaluate the ability of miR-1-3p expression (cutoff: 1.3) to discriminate between deeply and superficially invasive tumors. ROC analysis yielded an AUC of 0.729 (standard error = 0.116) with a wide confidence interval (95% CI: 0.502–0.957; *P* = 0.048).

When survival outcomes were analyzed, no association was observed between miR-1-3p expression and overall survival (OS; log-rank *P* = 0.854), with estimated 5-year OS rates of approximately 62% for patients with lower miR-1-3p expression (same cutoff of 1.3) and 68% for those with higher expression (Fig. 5C). Disease-free survival (DFS) analysis also showed a non-significant difference between groups (log-rank *P* = 0.097). Notably, no recurrence events were observed in the high-expression group (Fig. 5D).

## Discussion

Our study provides an integrative evaluation of the association between miR-1-3p expression and tumor invasiveness in OSCC across multiple experimental platforms. Functional *in vitro* assays showed that miR-1-3p inhibition significantly enhanced the invasive behavior of tumor cells. These findings were supported by *in vivo* analyses using a zebrafish xenograft model, in which miR-1-3p downregulation led to an increased tumor area and deeper infiltration relative to the control. In human tumor samples, a significant inverse correlation was observed between miR-1-3p expression and the depth of invasion, which was further reinforced by linear regression analysis. Additionally, deeply invasive tumors exhibited markedly lower levels of miR-1-3p than superficial tumors. Furthermore, survival analyses suggested a potential association between lower miR-1-3p expression and worse disease-free survival, although this finding did not reach statistical significance and should be interpreted with caution. Our findings expand prior evidence by demonstrating consistent phenotypic and translational associations across *in vitro*, *in vivo*, and human tumor analyses. These results suggested a potential role for miR-1-3p downregulation in tumor invasiveness, although this interpretation should be considered in light of the experimental limitations described. Moreover, these convergent findings support an association between miR-1-3p downregulation and a more aggressive phenotype characterized by increased invasiveness in OSCC, further highlighting the potential translational relevance of an integrative, multi-model approach combining experimental data with observations in human tumor samples.

Several studies have demonstrated that miR-1-3p acts as a tumor suppressor in OSCC and is consistently downregulated in tumor tissues and cell lines. Functional investigations have shown that its inhibition promotes proliferation, migration, and invasion, whereas its overexpression leads to a partial or complete reversal of these phenotypes. Prior studies have identified potential molecular targets of miR-1-3p, including Slug, DKK1, and PFN2, suggesting its involvement in pathways related to epithelial–mesenchymal transition and Wnt/β-catenin signaling [25–27]. However, these mechanisms were not directly investigated in the present study and are discussed here solely to provide biological context based on previously published data. Therefore, no mechanistic inferences can be drawn from the current dataset regarding these pathways.

The current study expands this understanding by providing a comprehensive functional characterization of miR-1-3p’s role in OSCC using complementary *in vitro*, *in vivo*, and *ex vivo* analyses. Our findings confirm that miR-1-3p inhibition significantly increases invasion *in vitro* and provides additional evidence suggesting that this effect may translate into larger and deeper tumors *in vivo*, as shown in a zebrafish xenograft model. Importantly, we also showed that miR-1-3p expression is inversely correlated with the depth of invasion in human tumor samples, and that deeply invasive tumors exhibit significantly lower levels of miR-1-3p, suggesting a biological association between miR-1-3p repression and a more aggressive tumor phenotype. Furthermore, while previous studies [8, 28] have pointed to network-level interactions and tumor–stromal crosstalk involving miR-1, our findings integrated these dimensions through multi-model validation. By confirming that miR-1-3p suppression not only enhances local invasion, but also increases tumor dissemination and metastatic spread *in vivo*, we futher support its potential relevance as a phenotypic regulator and as a candidate marker associated with tumor progression and dissemination.

Recent evidence from translational studies and reviews [29, 30] has emphasized the early and epigenetically driven suppression of miR-1 as a key event in oral oncogenesis. Our results are in agreement with these findings and provide additional support for the hypothesis that miR-1-3p downregulation may represent an active contributor to the acquisition of invasive traits.

Despite the integrative approach adopted in this study, several limitations of this study should be acknowledged. First, although the *in vivo* zebrafish model offers valuable insights into tumor behavior in living organisms, it does not fully recapitulate the complexity of the human tumor microenvironment, including immune modulation and stromal interactions. In addition, the zebrafish larval model lacks a fully developed adaptive immune system, and therefore tumor–immune interactions cannot be adequately assessed. This is particularly relevant given the growing evidence that tumor–microenvironment interactions, including immune cell infiltration and metabolic regulation, play a central role in the invasive behavior of squamous cell carcinomas [31, 32]. Furthermore, metastatic behavior observed in this model may not directly translate to human disease, and should be interpreted as supportive rather than definitive evidence of tumor aggressiveness. Additionally, most *in vitro* experiments were performed in duplicate, which is below the conventional standard of triplicate measurements in biological assays. Although consistent trends were observed across independent experiments, this limitation may affect the robustness of the quantitative estimates and should be considered when interpreting the experimental findings.

Second, while the study demonstrated consistent phenotypic effects following miR-1-3p inhibition, the gain-of-function experiments did not demonstrate a detectable inhibitory effect on tumor invasion or growth. This is reflected by the non-significant results observed in both the invasion assay (*P* = 0.068) and the zebrafish tumor size analysis (*P* = 0.784). Importantly, these findings indicate that miR-1-3p overexpression, under the conditions tested, did not produce a measurable phenotypic effect in these models. While the absence of a significant effect could be influenced by factors such as RNA interference machinery saturation, variability in transfection efficiency, or compensatory signaling pathways, these explanations remain speculative in the context of the present study, as no direct measurements of transfection efficiency or intracellular miR-1-3p levels were performed. In particular, as reported in previous studies, supraphysiological levels of microRNA mimics may lead to saturation of the RNA interference machinery, including competition for Argonaute proteins and other components of the RISC complex, resulting in non-physiological or attenuated functional effects. This phenomenon has been previously described [33, 34], where excessive levels of small RNAs can interfere with endogenous microRNA processing and function, potentially obscuring true biological effects. Therefore, these findings should be interpreted with caution when inferring a functional role of miR-1-3p in tumor invasion based on gain-of-function experiments. Additionally, the identification of direct molecular targets was beyond the scope of this study, and future studies should aim to validate the downstream effectors mediating the invasive phenotype observed. No pathway analysis or functional rescue experiments were performed, and therefore the molecular mechanisms underlying the observed phenotypic effects remain to be elucidated.

Moreover, the sample size for the *ex vivo* analysis was relatively small, which limits the generalizability of the observed correlation between miR-1-3p expression and the depth of invasion. Therefore, the clinical findings should be interpreted as preliminary and exploratory, rather than as evidence of clinical applicability. In addition, this cohort was retrospectively assembled, and the stratification of tumors according to depth of invasion was based on predefined cutoff values (5, 15, and 25 mm), selected to represent distinct invasion profiles while maintaining feasibility of analysis from our cohort. However, this approach may not fully capture the continuous biological spectrum of tumor invasion. Furthermore, no adjustment for potential confounding variables was performed, which should be considered when interpreting these findings. In addition, given the exploratory nature of the study, no formal correction for multiple comparisons was applied, and therefore the statistical significance of some findings should be interpreted with caution. Accordingly, statistical findings should be considered exploratory rather than confirmatory, with emphasis on concordance across models rather than on individual P-values. Another important limitation is that the reduced size of the validation cohort likely limited the statistical power of the survival analyses, which should be considered underpowered and therefore interpreted as inconclusive, and may have contributed to the absence of significance in OS, as well as a potential overestimation of DFS differences, particularly given that no recurrence events were observed among patients with higher miR-1-3p expression during follow-up, introducing a floor effect that further limits interpretability, limiting the interpretation of these findings in a translational context. Similarly, although the ROC analysis reached nominal statistical significance, the wide confidence interval, spanning values close to chance level, indicates substantial imprecision and does not support conclusions regarding discriminative performance or clinical utility. Finally, although multiple levels of evidence support the role of miR-1-3p downregulation in promoting tumor aggressiveness, this is still a cross-sectional analysis; causal inference in clinical settings would benefit from prospective studies and longitudinal tissue sampling.

In conclusion, this study provides exploratory evidence supporting an association between miR-1-3p downregulation and increased invasive tumor behavior in OSCC, within the constraints of the experimental design. The consistent findings across *in vitro* assays, *in vivo* zebrafish models, and *ex vivo* analysis of human tumor samples support a role for miR-1-3p in tumor invasiveness and suggest its potential relevance for future evaluation as a biomarker candidate, particularly in relation to depth of invasion. This integrative methodological design highlights the translational relevance of combining experimental and clinical data and provides a framework for further investigation. Future studies should aim to identify and functionally validate the downstream targets of miR-1-3p in the invasive phenotype, explore the epigenetic mechanisms underlying its silencing, and assess its value as a predictive or prognostic biomarker in larger prospective patient cohorts. In addition, further studies are needed to determine whether modulation of miR-1-3p expression may have therapeutic implications in OSCC.

## Data Availability

No datasets were generated or analysed during the current study.

## Code availability

The data supporting the findings of this study are available from the corresponding author, LLM, upon reasonable request.

## Declarations

### Generative AI in scientific writing

The authors declare that AI- and AI-assisted technologies in the writing process were not used for the preparation of this study.
